# Asparaginyl-tRNA Synthetase, a Novel Component of Hippo Signaling, Binds to Salvador and Enhances Yorkie-Mediated Tumorigenesis

**DOI:** 10.3389/fcell.2020.00032

**Published:** 2020-02-05

**Authors:** Eunbyul Yeom, Dae-Woo Kwon, Jaemin Lee, Seok-Ho Kim, Ji-Hyeon Lee, Kyung-Jin Min, Kyu-Sun Lee, Kweon Yu

**Affiliations:** ^1^Metabolism and Neurophysiology Research Group, Korea Research Institute of Bioscience and Biotechnology, Daejeon, South Korea; ^2^Tunneling Nanotube Research Center, Korea University, Seoul, South Korea; ^3^Department of Functional Genomics, University of Science and Technology, Daejeon, South Korea; ^4^Industrial Bio-materials Research Center, Korea Research Institute of Bioscience and Biotechnology, Daejeon, South Korea; ^5^Department of Medicinal Biotechnology, College of Health Sciences, Dong-A University, Busan, South Korea; ^6^Department of Biological Sciences, Inha University, Incheon, South Korea; ^7^Convergence Research Center of Dementia, Korea Institute of Science and Technology, Seoul, South Korea

**Keywords:** asparaginyl-tRNA synthetase (NRS), Salvador, Hippo signaling, tirandamycin B, tumorigenesis

## Abstract

Aminoacyl-tRNA synthetases (ARSs), which are essential for protein translation, were recently shown to have non-translational functions in various pathological conditions including cancer. However, the molecular mechanism underlying the role of ARSs in cancer remains unknown. Here, we demonstrate that asparaginyl-tRNA synthetase (NRS) regulates Yorkie-mediated tumorigenesis by binding to the Hippo pathway component Salvador. *NRS-RNAi* and the NRS inhibitor tirandamycin B (TirB) suppressed Yorkie-mediated tumor phenotypes in *Drosophila*. Genetic analysis showed that *NRS* interacted with Salvador, and *NRS* activated Hippo target genes by regulating Yorkie phosphorylation. Biochemical analyses showed that NRS blocked Salvador-Hippo binding by interacting directly with Salvador, and TirB treatment inhibited NRS-Salvador binding. YAP target genes were upregulated in a mammalian cancer cell line with high expression of NRS, whereas TirB treatment suppressed cancer cell proliferation. These results indicate that NRS regulates tumor growth by interacting with Salvador in the Hippo signaling pathway.

## Introduction

Aminoacyl-tRNA synthetases (ARSs) are enzymes that catalyze the binding of amino acids to their cognate tRNAs with high fidelity (McClain, 1993; Delarue, 1995; Ogunbiyi et al., 1998). Once the tRNA is charged, ribosomes transfer the amino acid from the tRNA onto a growing peptide according to the specific genetic code (McClain, 1993; Delarue, 1995; Ogunbiyi et al., 1998). ARSs play an important role in the translation of RNA into protein. In addition to their canonical role in protein translation, ARSs function in metabolism, development, inflammatory responses, and tumorigenesis (Park et al., 2008). ARSs are composed of a highly conserved catalytic domain and an RNA-binding domain that recognizes tRNA anticodons (Arnez and Moras, 1997). In addition to these two domains, certain ARSs contain an amino acid-binding pocket that is essential for non-translational functions (Guo and Schimmel, 2013). The non-translational functions of ARSs have been expanded by the identification of new domains involved in protein-protein interactions (PPIs).

Aminoacyl-tRNA synthetases are involved in cancer cell survival and tumor progression. Low expression of tryptophanyl-tRNA synthetase (WRS) in colorectal cancer correlates with increased risk of metastasis and poor prognosis (Ghanipour et al., 2009). Nine ARSs in species from *Drosophila* to mammals [glutamyl-prolyl-tRNA synthetase, isoleucyl-tRNA synthetase, leucyl-tRNA synthetase, glutaminyl-tRNA synthetase (QRS), lysyl-tRNA synthetase (KRS), arginyl-tRNA synthetase, aspartyl-tRNA synthetase, and methionyl-tRNA synthetase] can form macromolecular multi-synthetase complexes (MSCs) with three scaffold proteins (Cerini et al., 1991; Quevillon and Mirande, 1996; Rho et al., 1999). Among them, KRS is involved in the development of melanoma (Levy and Fisher, 2011), and QRS inhibits apoptosis (Ko et al., 2001). A recent study suggests that methionyl-tRNA synthetase is overexpressed in non-small cell lung cancer (Kim et al., 2017). Although these ARSs have potential roles in tumorigenesis, the molecular mechanism underlying the role of ARSs in cancer remains unclear.

In this study, we used a *Drosophila* model system to identify candidate ARSs involved in tumorigenesis, and analyzed the tumorigenic effects of ARSs to elucidate the underlying molecular mechanisms. *Drosophila* is an excellent genetic model system because it has several conserved signaling pathways that are also present in mammals. *Drosophila* is therefore a powerful model to study human disorders such as cancer and neurodegenerative diseases. The Hippo (Hpo) signaling pathway is a well-studied pathway involved in growth regulation. The Hpo signaling pathway is evolutionary conserved from *Drosophila* to mammals, and evidence indicates that dysregulation of the Hpo pathway is involved in many types of human cancer (Halder and Johnson, 2011; Yu et al., 2015; Zanconato et al., 2016). The core components of the Hpo pathway are the Hpo/Mst1/2 (Harvey et al., 2003; Jia et al., 2003; Pantalacci et al., 2003; Udan et al., 2003; Wu et al., 2003) kinase, the scaffold protein Salvador (Sav/SAV1) (Kango-Singh et al., 2002; Tapon et al., 2002), and the Warts (Wts/Lats1/2) (Justice et al., 1995; Xu et al., 1995) kinase. The activated Hpo-Sav complex phosphorylates and activates the downstream Wts (Harvey et al., 2003; Wu et al., 2003), which phosphorylates and inactivates the transcription factor Yorkie (Yki/YAP) (Huang et al., 2005; Zhao et al., 2007). Inhibition of the core kinase cascade results in the translocation of unphosphorylated Yki to the nucleus, where it activates the expression of target genes of the Hpo pathway that regulate cell proliferation and survival (Huang et al., 2005).

Here, we established a fly cancer model by overexpressing Yki to investigate the tumorigenic effect of ARSs. The results showed that asparaginyl-tRNA synthetase (NRS) is highly expressed in cancer, and physically and genetically interacts with Salvador, a Hpo pathway component. NRS inhibited Sav-Hpo binding by sequestering Sav, and activated the expression of Yki target genes. Inhibition of NRS rescued the tumor phenotype in *Drosophila* and mammalian cancer cells. The present results demonstrate that NRS is a conserved growth regulator that modulates Hpo signaling.

## Materials and Methods

### *Drosophila* Genetics and Transgenes

*Drosophila melanogaster* was maintained at 25°C on standard media. *GMR-Gal4*, *UAS-yki^*WT*^-GFP*, and *UAS-yki^*S*168*A*^-GFP* were obtained from the Bloomington Stock Center (Bloomington, IN, United States), and all *Drosophila ARS-RNAi* lines were obtained from Vienna *Drosophila* RNAi Center (VDRC, Vienna, Austria). *UAS-Sav* was a gift from Nicholas Tapon (The Francis Crick Institute, United Kingdom), and *en-Gal4-GFP* was a gift from Kwang-Wook Choi (KAIST, South Korea). The *pUAST-NRS* transgenic fly was generated by p-element-mediated germline transformation (Rubin and Spradling, 1982).

### Supplementation With Tirandamycin B

Tirandamycin B (TirB) (ChemFaces, Hubei, China) solution was added to the standard medium during food preparation to a final TirB concentration of 0, 20, or 50 μM. TirB-supplemented food (3 mL) at the indicated concentrations was delivered to individual *Drosophila* polypropylene vials, and the flies were cultured.

### Cell Culture

The LLC and C26 cell lines were obtained directly from the ATCC and cultured according to the manufacturer’s guidelines. LLC and C26 cells were purchased in 2018. These cell lines were periodically authenticated by monitoring cell morphology, growth curve analysis, and inspection of Mycoplasma contamination using a Mycoplasma detection kit (Lonza). Cells were cultured at 37°C in a humidified 5% CO_2_ incubator in Dulbecco’s modified Eagle’s medium containing 10% fetal bovine serum (FBS). Cells transfected with YAP-siRNA were considered as the siRNA group (siYAP) (siYAP-1: CUGCUAUGAUAACUACGUU, siYAP-2: CCGGCUCUAAAGAACCCGA), cells transfected with scrambled-siRNA were considered to be the negative control group (siScr), and cells without any treatment were considered to be the blank control group (cont).

### WST-1 Proliferation Assay

The WST-1 measurement was performed according to the manufacturer’s standard protocol. LLC and C26 cells (1 × 10^4^/well) were cultured in 96-well plates and incubated overnight. TirB was added to a final concentration of 10 ng/mL and incubated for 24, 48, and 72 h. After addition of 10 μL of WST-1, cells were incubated for an additional 2 h at 37°C. The absorbance was monitored at 450 nm.

### Lifespan Assays

This experiment used the *CS*_10_ (*w*^1118^ outcrossed 10 times to Canton-S) *D. melanogaster* strain, which was obtained from the Bloomington Drosophila Stock Center at Indiana University. Flies were reared at 25°C and 65% humidity on a 12:12 h light:dark cycle. Fly larvae were reared in standard cornmeal-sugar-yeast with agar (CSY) medium (5.2% cornmeal, 11% sugar, 2.6% baker’s yeast, 0.5% propionic acid, 0.04% methyl-4-hydroxybenzoate, and 0.8% agar). Newly eclosed male and female adult fruit flies (100 of each) were transferred to a 500 cm^2^ demography cage. Three replicate cages were set up for each experimental group. Vials containing fresh SY food with/without TirB were affixed to the cages and changed every 2 days, at which time dead flies were removed and recorded.

### Immunostaining

For immunostaining, larval discs were dissected in PBS, fixed in 4% paraformaldehyde, blocked in 5% BSA, and incubated in primary antibodies overnight at 4°C, followed by secondary antibody incubation for 2 h at room temperature. The tissues were mounted with Vectashield mounting medium (Vectashield), and fluorescence images were acquired with a FluoView confocal microscope (Olympus). Primary antibodies used were rabbit anti-phospho-histone3 (Santa Cruz Biotechnology, Santa Cruz, CA, United States; 1:200), mouse anti-CD2 (AbD Serotec, 1:200), rabbit anti-CycE (Santa Cruz Biotechnology, 1:200), and goat anti-Diap1 (Santa Cruz Biotechnology, 1:200); and secondary antibodies were anti-mouse IgG Alexa 488, anti-rabbit IgG Alexa 594, and anti-goat IgG Alexa 594 (Life Technologies, 1:200).

### Transfection, Immunoprecipitation, and Western Blot Analysis

*Drosophila* S2 cells were cultured in Schneider’s Insect media (Sigma) with 10% FBS (Gibco). Transfection was carried out with Effectene reagent (Qiagen) according to the manufacturer’s instructions. Each transfection was performed using 1–2 μg of DNA. For the immunoprecipitation assay, cells were lysed on ice for 30 min with lysis buffer containing 1 M Tris pH 7.4, 5 M NaCl, 0.5 M EDTA, 0.1% NP-40, and 5% glycerol. The cell lysates were incubated with Anti-FLAG M2 magnetic beads (Sigma) overnight at 4°C. Immunoprecipitates were washed three times with lysis buffer and subjected to SDS-PAGE. Western blot analysis was performed using a standard protocol. Yki phosphorylation was examined using a 12.5% acrylamide phos-tag gel (50 μM) (Wako). Primary antibodies used were mouse anti-HA (Invitrogen, 1:5000), rabbit anti-Sav (Dr. Jiang, 1:2000) (Yue et al., 2012), rabbit anti-Yki (Dr. Irvine, 1:4000) (Oh and Irvine, 2008), mouse anti-V5 (Invitrogen, 1:5000), mouse anti-Myc (Santa Cruz Biotechnology, 1:1000), guinea pig anti-Hpo (Dr. Halder, 1:2000), rabbit anti-pS6K (Invitrogen, 1:1000), and mouse β-actin (1:3000, DSHB). Secondary antibodies were anti-rabbit IgG (Santa Cruz Biotechnology, 1:5000), anti-mouse IgG (Pierce, 1:5000), and anti-guinea pig IgG (Jackson, 1:5000). Western blot bands were quantified using ImageJ software.

### RNA Preparation and Quantitative Real-Time PCR

Total RNA was extracted using the easy-BLUE^TM^ reagent (iNtRON Biotechnology). All RNA samples were treated with RNase-free DNase (Promega), and cDNA was synthesized using a SuperScript III First-Strand Synthesis System (Invitrogen). Quantitative RT-PCR analysis was performed using an ABI Prism 7900 Sequence Detection System (Applied Biosystems) and SYBR Green PCR Core reagents (Applied Biosystems). Data were analyzed using the comparative cycle threshold (Ct) method (User Bulletin 2, Applied Biosystems). All experiments were repeated at least three times, and the data are presented as the mean and error bar (±SEM).

### Statistics

All statistical significance were tested by Microsoft Excel-based application for the Student’s *t*-test statistical analysis. Values in this paper are presented as the mean ± SEM *p*-values were as follows: ^∗^*p* < 0.05; ^∗∗^*p* < 0.01; ^∗∗∗^*p* < 0.001.

## Results

### Knockdown of *NRS* Suppresses *yki*-Induced Tumor Phenotypes in the Adult Eye and Larval Eye Disc

**FIGURE 1:**
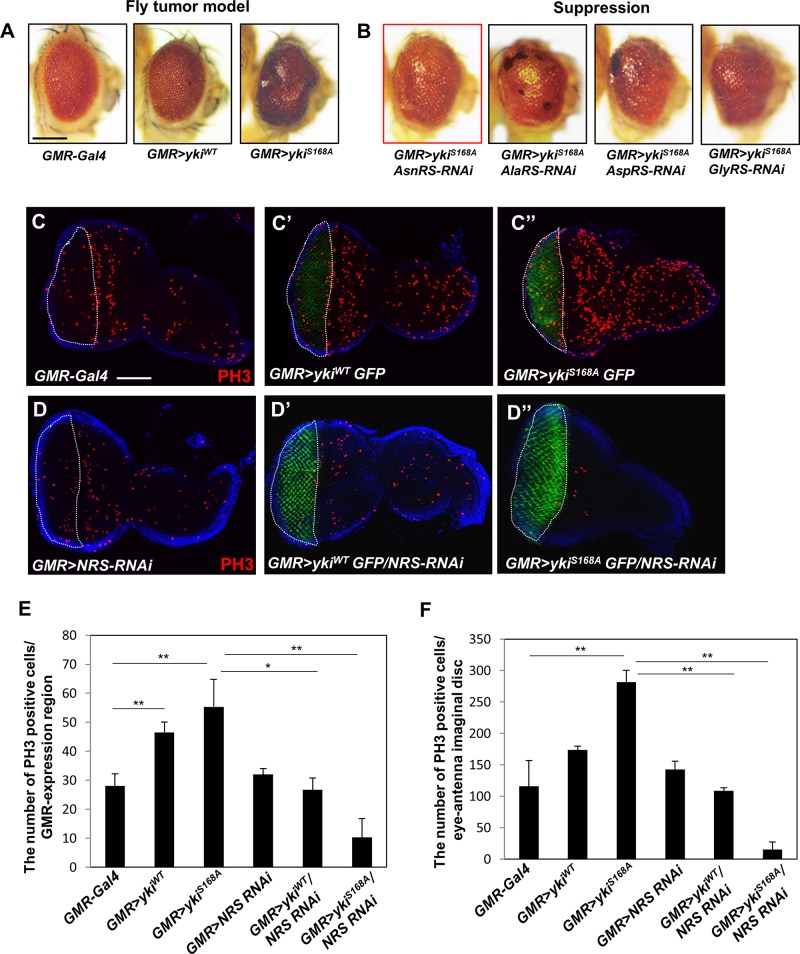
Knockdown of *NRS* suppresses the *yki*-induced tumor phenotype in the adult eye and larval eye disc. **(A)** Overexpression of the wild-type form of *yki* caused mild roughness and overexpression of the active form of *yki* caused abnormal overgrowth of the eye. **(B)** Library screening showed that *AsnRS-RNAi* strongly suppressed the eye phenotype induced by *yki* overexpression. Scale bar, 200 μm. **(C–C”)** Overexpression of *yki*^*WT*^ and *yki^*S*168*A*^* in the larval eye disc increased cell proliferation, as determined by detection of phospho-H3 expression in the GMR-expressing region compared with the control. **(D–D”)** Consistent with the adult eye phenotype, knockdown of NRS significantly rescued cell proliferation caused by *yki* overexpression. Blue, DAPI; green, GFP; red, phospho-H3. Scale bar, 50 μm. **(E)** Quantification of phospho-H3-positive cells in the GMR-expressing region [white-dashed line in panels **(C,D)**]. **(F)** Quantification of phospho-H3-positive cells in the eye-antenna disc. (*GMR-Gal4 n* = 6, *GMR* > *yki*^*WT*^ *n* = 7, *GMR* > *yki*^*SA*^ *n* = 9, *GMR* > *NRS RNAi n* = 6, *GMR* > *yki^*WT*^/NRS RNAi n* = 8, *GMR* > *yki^*S*168*A*^/NRS RNAi n* = 5). Data are presented as the mean ± SEM. *p < 0.05, **p < 0.01.

To identify candidate ARSs involved in growth regulation, we first established a *Drosophila* cancer model by overexpressing *yki*, a downstream transcription factor of the Hpo pathway, in the adult eye using *GMR-Gal4*. Expression of wild-type *yki* (*GMR* > *yki*^*WT*^) caused mild rough eye phenotype (Figure 1A, middle panel), whereas the constitutively active form of *yki* (*GMR* > *yki^*S*168*A*^*) resulted in abnormal overgrowth of the eye by inducing massive cell proliferation (Figure 1A, right panel). *GMR* > *yki^*S*168*A*^* flies were crossed with each ARS-RNAi line, and the anticancer effect by knockdown of ARSs was analyzed in the *Drosophila* cancer model. Nine *ARS-RNAi* lines (*ArgRS*, *CysRS*, *GlnRS*, *GluproRS*, *HisRS*, *LysRS*, *ThrRS*, *TyrRS*, and *SerRS*) showed pupal lethality, indicating a deleterious effect on survival in the *yki*-induced tumor model. Five *ARS-RNAi* lines (*LeuRS*, *MetRS*, *PheRS*, *TrpRS*, and *VarRS*) showed no significant changes (Supplementary Figure S1A), whereas the *AsnRS*-, *AlaRS*-, *AspRS*-, and *GlyRS-RNAi* lines showed suppression of the overgrowth tumor phenotype (Figure 1B). Among them, *Asn-tRNA synthetase* (*NRS*) knockdown most effectively suppressed the *yki*-overexpression phenotype in the adult eye (Figure 1B, red box). We then tested whether NRS expression was altered in cancer, and the results showed significant upregulation of *Drosophila* NRS in the *yki*-overexpression background (Supplementary Figure S1B).

To confirm that NRS knockdown suppressed the *yki*-induced tumor phenotype, we performed immunostaining of the larval eye disc using the mitotic marker phospho-H3. Overexpression of *yki* markedly increased cell proliferation in the GMR-expressing region marked by GFP (Figures 1C–C”,E,F). Consistent with the results of the adult eye phenotype, NRS knockdown by *NRS-RNAi* significantly suppressed cell proliferation induced by *yki* overexpression (Figures 1D–D”,E–F). As the control, KRS knockdown had no significant effect on cell proliferation (Supplementary Figures S1C,D), suggesting that the suppression of the tumor phenotype was NRS-specific.

### The NRS Inhibitor Tirandamycin B Suppresses *yki*-Induced Tumor Phenotypes

**FIGURE 2:**
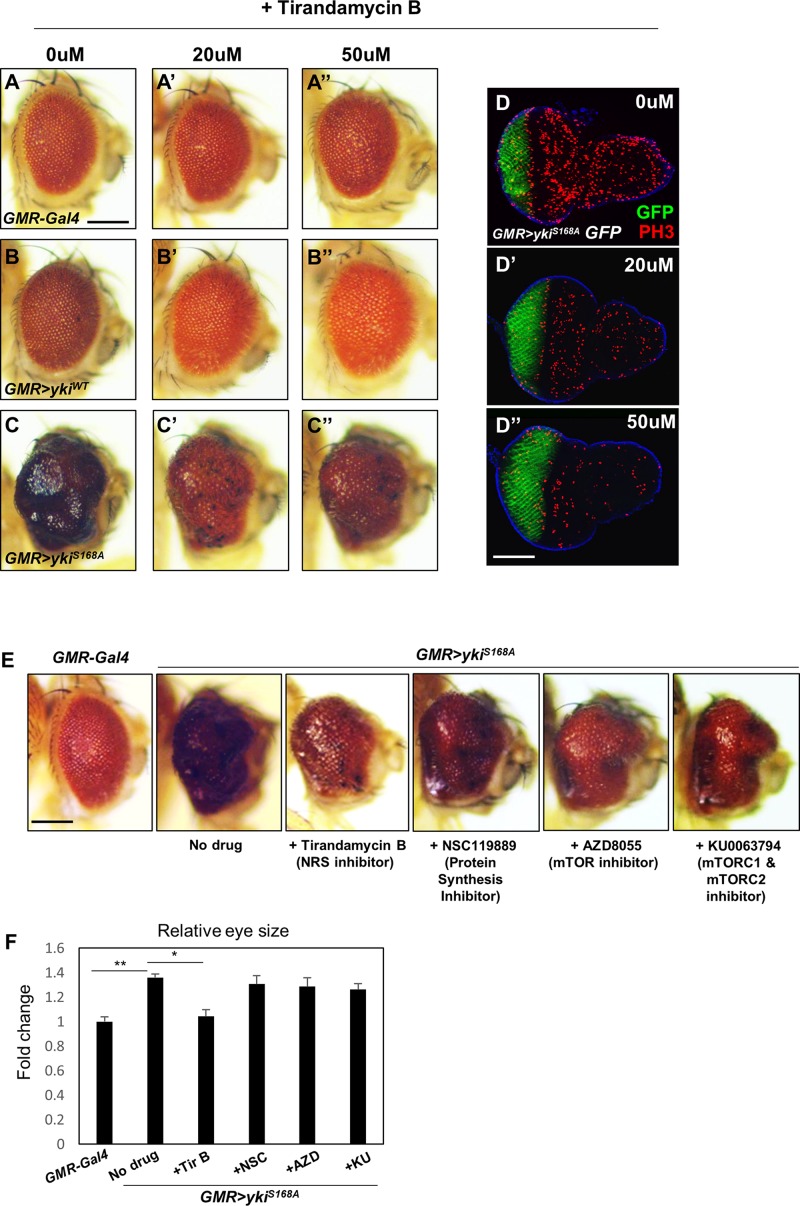
The NRS inhibitor tirandamycin B (TirB) suppresses the *yki*-induced tumor phenotype. **(A–C)** TirB treatments dose-dependently rescued the tumor growth phenotypes induced by *yki* overexpression. **(A–A”)** *GMR-Gal4* control flies were treated with 0, 20, or 50 μM TirB. **(B–B”)** *GMR* > *yki*^*WT*^ flies were treated with 0, 20, or 50 μM TirB. **(C–C”)** *GMR* > *yki^*S*168*A*^* flies were treated with 0, 20, or 50 μM TirB. Scale bar, 200 μm. **(D)** TirB decreased cell proliferation detected by phospho-H3 expression in the larval eye disc of *GMR* > *yki^*S*168*A*^* flies. Blue, DAPI; green, GFP; red, phospho-H3. Scale bar, 50 μm. **(E)** Inhibitors blocking protein synthesis or translation do not suppress the *yki*-mediated tumor phenotype. Scale bar, 200 μm. **(F)** Quantification of relative eye size of panel **(E)**. Data are presented as the mean ± SEM. *p < 0.05, **p < 0.01.

To test the effect of chemical inhibition of NRS on the *yki*-induced tumor phenotype, *GMR* > *yki^*S*168*A*^* flies were treated with the NRS inhibitor TirB, which was originally isolated from natural products and identified as an anti-filarial drug targeting *Brugia malayi* NRS (Yu et al., 2011). TirB treatment suppressed the *yki*-overexpression phenotype in a dose-dependent manner. TirB at 50 μM effectively rescued the tumor phenotype (Figures 2A–C). In the larval eye discs, TirB also dose-dependently suppressed cell proliferation, as determined by detection of the phospho-H3 marker (Figures 2D–D”). TirB treatment was not cytotoxic to either male or female flies at 10, 20, or 50 μM. TirB had no obvious effect on fly lifespan even at 50 μM, an effective concentration for tumor inhibition (Supplementary Figures S2A,B). Since, NRS functions mainly in protein translation, we tested whether NRS knockdown affected translation in our cancer model. NRS knockdown partially restored the increased phosphorylation of S6K induced by *yki* overexpression (Supplementary Figures S2C,D). To determine whether the rescue effect was caused by inhibition of protein translation, we tested the effect of inhibitors of protein synthesis or translation. Among inhibitors we tested, TirB strongly rescued the eye size of *GMR* > *yki^*S*168*A*^* flies (Figures 2E,F). These data suggest that the NRS inhibitor TirB has a specific anticancer effect in the *Drosophila* tumor model.

### NRS Genetically Regulates the Hpo Pathway Component Sav

**FIGURE 3:**
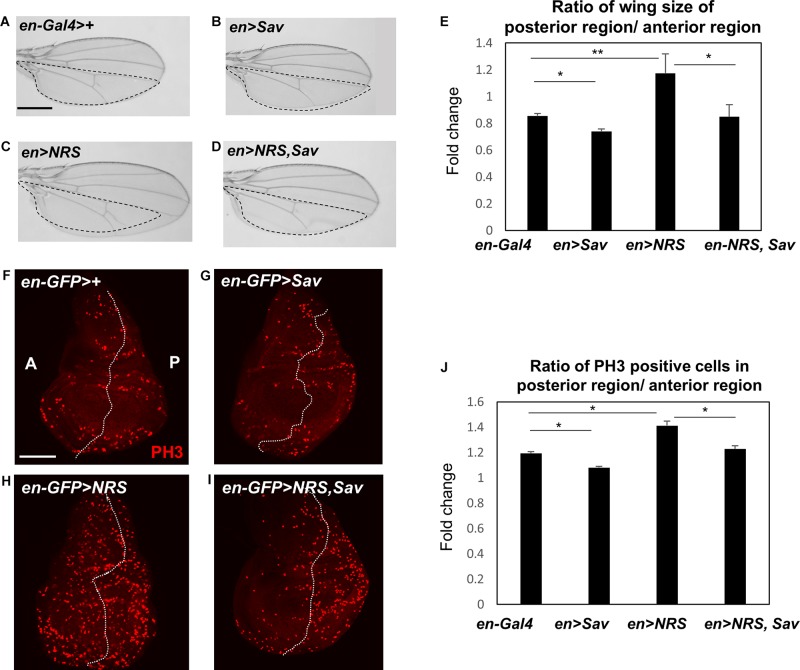
NRS genetically regulates the Hippo pathway component Salvador. **(A)** *en-Gal4*>+, the control wing (*n* = 7). **(B)** Overexpression of Salvador (*Sav*) (*en* > *Sav*) resulted in a slightly smaller wing (*n* = 9). **(C)** Overexpression of *NRS* (*en* > *NRS*) resulted in a larger wing (*n* = 10). **(D)** Co-expression of *NRS* and *Sav* (*en* > *NRS, Sav*) rescued the enlarged wing size induced by *NRS* overexpression (*n* = 9). Scale bar, 100 μm. **(E)** Quantification of relative wing size from panels **(A–D)**. **(F)** *en-GFP* > +, the control wing disc. **(G)** *Sav* overexpression (*en-GFP* > *Sav*) in the wing disc decreased cell proliferation. **(H)** Overexpression of *NRS* (*en-GFP* > *NRS*) induced a dramatic increase of cell proliferation in the posterior wing disc. **(I)** *NRS*-induced cell proliferation was rescued by addition of *Sav* (*en-GFP* > *NRS, Sav*). Scale bar, 50 μm. **(J)** Quantification of PH3-positive cells in the posterior wing disc. Data are presented as the mean ± SEM. *p < 0.05, **p < 0.01.

The effect of NRS knockdown on rescuing the *yki*-mediated tumor phenotype led us to hypothesize that NRS may interact with a Hpo signaling component. Screening of the database of protein interactions in *Flybase* (yeast two-hybrid screening from Curagen) (Giot et al., 2003) identified Sav as a potential NRS-interacting protein. We first examined the genetic interaction between *NRS* and *Sav*. Overexpression of *Sav* by *en-Gal4* in the adult wing resulted in the formation of slightly smaller wings (Figures 3B,E), whereas *NRS* overexpression led to increased wing size (Figures 3C,E) compared with that of the *en* > + control (Figure 3A). The enlarged wing phenotype induced by *NRS* overexpression was rescued by overexpression of *Sav* (Figures 3D,E). The genetic interaction was confirmed in the larval wing disc by immunostaining for the PH3 mitotic cell marker. Consistently, overexpression of *Sav* and *NRS* decreased (Figures 3G,J) and increased (Figures 3H,J) the number of PH3-positive mitotic cells, respectively, in the posterior compartment compared with that in the anterior control compartment (Figures 3F,J). Co-expression of *Sav* and *NRS* slightly rescued the *NRS* phenotype (Figures 3I,J). The effect of *Sav* and *NRS* overexpression was tested in the adult eye using *eyeless-Gal4*, and the results were similar to those observed in the wing (Supplementary Figure S3). These genetic interactions suggested that NRS regulates Sav.

### NRS Overexpression Activates Hpo Pathway Target Genes

**FIGURE 4:**
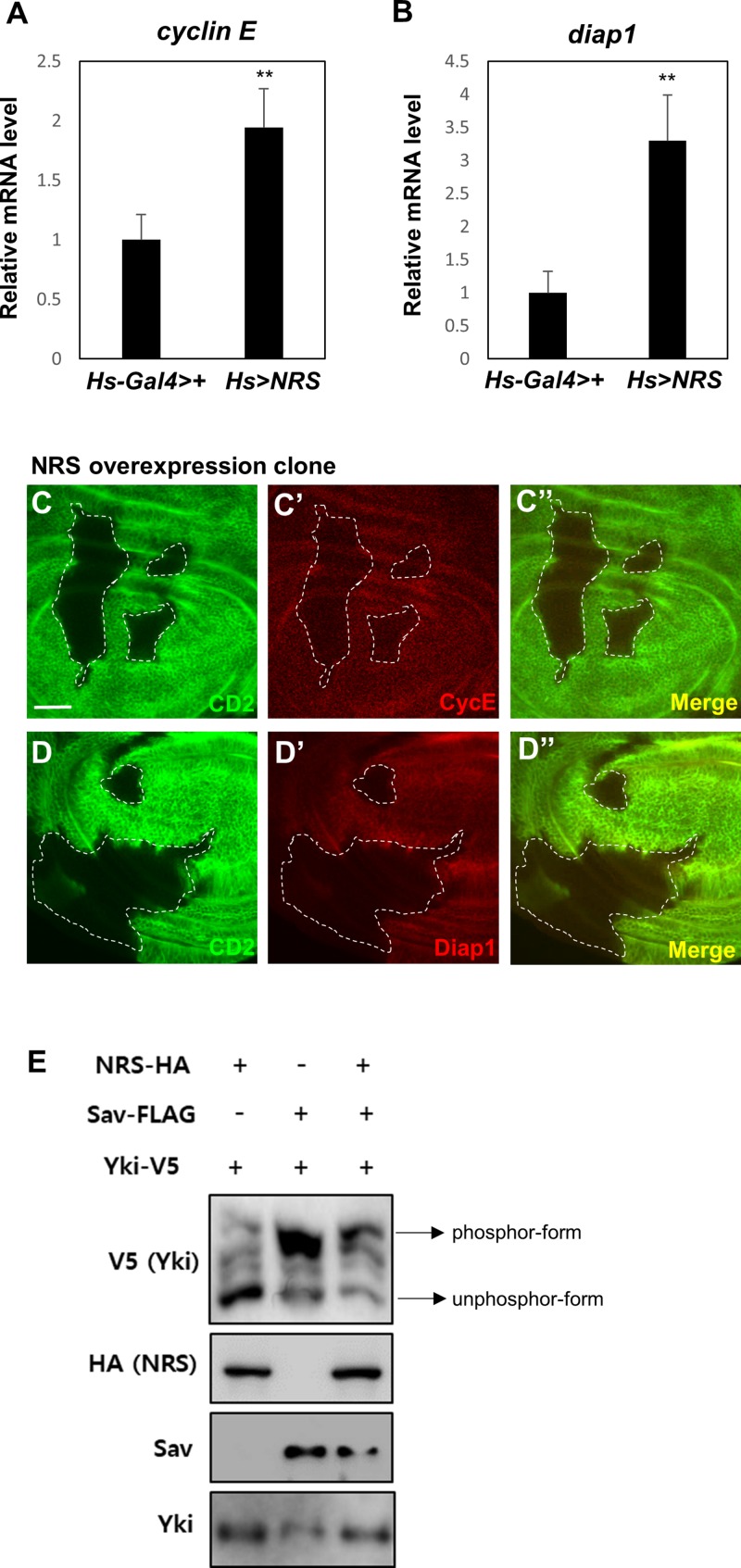
NRS overexpression activates Hippo pathway target genes. Adult whole body *NRS* overexpression (*Hs* > *NRS*) significantly upregulated *cyclin E* **(A)** and *diap1* **(B)** target gene expression. **(C,D)** Consistent with mRNA levels, the CycE and Diap1 proteins were upregulated in the *NRS*-overexpressing clone marked by membrane marker CD2. The genotype of the *NRS* overexpression clone was *yw hs-FLP*; *act* > *y* + > *Gal4 (Ay-Gal4) UAS-CD2/UAS-NRS*. Scale bar, 20 μm. **(E)** NRS overexpression decreased the phosphorylation level of Yki in S2 cells, as detected by the phos-tag gel. Data are presented as the mean ± SEM. **p < 0.01.

The genetic interactions between NRS and Sav suggested that NRS positively regulates Yki signaling. Downregulation of *Hpo* or *Sav* increases Yki activity and activates the transcription of Yki target genes such as *cyclin E* and *diap1*, promoting cell cycle progression and inhibiting cell death, respectively (Huang et al., 2005). We therefore examined the effect of *NRS* overexpression on the transcription of Yki target genes. NRS overexpression by *hs-Gal4* upregulated *cyclin E* and *diap1* mRNA expression (Figures 4A,B). *NRS* overexpression clones in wing discs upregulated Cyclin E and Diap1 protein expression (Figures 4C–C”,D–D”). Because upregulation of Hpo or Sav increases the phosphorylation of Yki and inhibits Yki nuclear localization (Oh and Irvine, 2008), we examined the effect of NRS on the phosphorylation of Yki. In S2 cells, Sav induced a mobility shift of V5 tag-Yki on a phos-tag gel (Nagy et al., 2018), and NRS co-expression decreased Yki phosphorylation (Figure 4E). These results indicate that NRS activates Yki target genes by regulating Yki phosphorylation.

### NRS Promotes the Dissociation of Hpo-Sav Binding by Interacting With Sav

**FIGURE 5:**
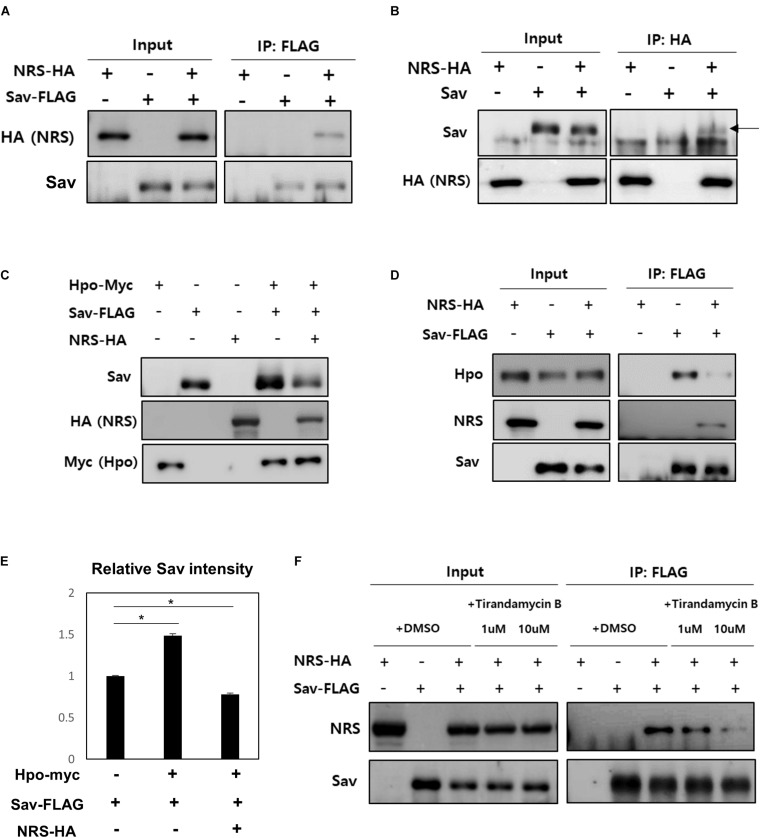
NRS promotes the dissociation of the Hippo-Salvador complex by interacting with Salvador. **(A)** Co-immunoprecipitation (Co-IP) assays showed that Sav physically interacts with NRS, as detected by Sav immunoprecipitation and NRS immunoblotting. **(B)** Co-IP between Sav and NRS was confirmed by immunoprecipitating NRS. **(C)** Hippo (Hpo) co-expression upregulated Sav expression, whereas Sav level was strongly reduced by addition of NRS. **(D)** Co-IP assays showed that binding of NRS to Sav dissociated the Sav-Hpo complex. **(E)** Quantification of Sav protein levels in panel **(C)**. **(F)** Direct binding of NRS to Sav was reduced by TirB treatment in a dose-dependent manner. Data are presented as the mean ± SEM. *p < 0.05.

To further understand the mechanism underlying the role of NRS in Hpo signaling-mediated growth regulation, the PPI of NRS with Hpo signaling components (Hpo, Sav, and Yki) was tested by co-immunoprecipitation assays in S2 cells. The results showed that NRS binds directly to Sav (Figures 5A,B), but not to Hpo (Supplementary Figure S3F) or Yki (Supplementary Figure S3G). Sav is a scaffold protein in which the stabilization effect depends on binding to Hpo (Pantalacci et al., 2003). We confirmed that Sav is stabilized when Hpo is co-transfected with Sav (Figure 5C, fourth lane, E). Addition of NRS significantly decreased the protein level of Sav (Figure 5C, fifth lane, E). This result suggests that NRS overexpression interferes with the interaction between Sav and Hpo, resulting in the destabilization of Sav. To test this hypothesis, we examined the binding of Sav and Hpo in cells co-transfected with NRS, which showed that the interaction between Sav and Hpo was significantly reduced (Figure 5D). This suggested that NRS acts as a competitive inhibitor of Hpo-Sav binding. To confirm this inhibition of Hpo-Sav binding by NRS regulates *yki^*S*168*A*^*-induced tumor growth, we showed that over-expression of Hpo suppressed the overgrowth eye tumor phenotype of *GMR* > *yki^*S*168*A*^* (Supplementary Figure S3I). The NRS inhibitor TirB effectively rescued tumor growth induced by *yki* overexpression in fly eyes in a dose-dependent manner (Figures 2C–C”). In S2 cells, TirB dose-dependently inhibited NRS-Sav binding and also partially restored the Sav level (Figure 5F and Supplementary Figure S3H). These results indicated that inhibition of NRS rescued tumor growth by blocking the interaction between NRS and Sav.

### NRS Overexpression Activates Hpo Signaling in Mammalian Cancer Cells

**FIGURE 6:**
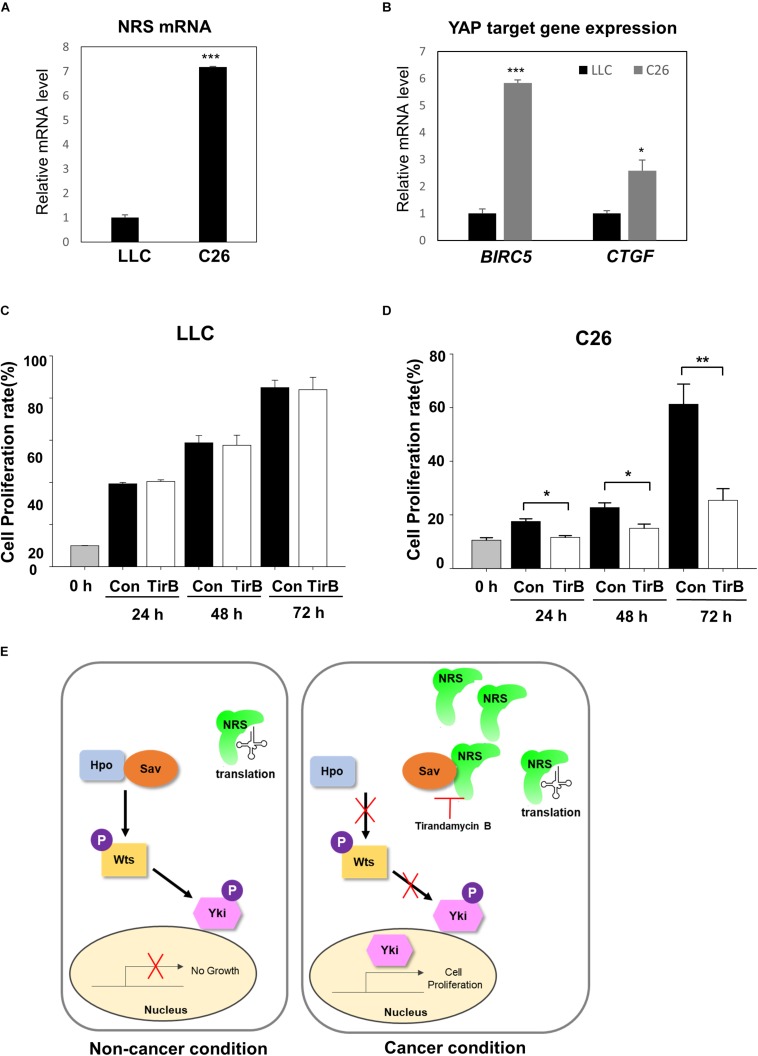
NRS-overexpressing mammalian cancer cells activate Hippo signaling. **(A)** *NRS* expression was higher in the C26 (colon carcinoma) cell line than in the LLC (Lewis lung carcinoma) cell line. **(B)** Expression of YAP target genes (*BIRC5* and *CTGF*) was significantly higher in C26 cells than in LLC cells. **(C,D)** Cell proliferation assays in cells treated with TirB showed that cell proliferation was strongly suppressed in the C26 cell line **(D)**, but not in the LLC cell line **(C)**. Data are presented as the mean ± SEM. *p < 0.05, **p < 0.01, ***p < 0.001. **(E)** Proposed model of NRS function: NRS has a canonical translational function in the non-cancer condition. In cancer, NRS is upregulated and binds to Sav, which inhibits the interaction between Sav and Hpo. This inhibits the Hpo-Wts-Yki phosphorylation cascade, leading to Yki translocation to the nucleus to activate target genes.

Next, we investigated the novel role of NRS in growth regulation through the Hpo pathway in mammalian cancer models. We used two mouse cancer cell lines, a Lewis lung carcinoma cell line (LLC) and a colon carcinoma cell line (C26). First, we checked the expression of *NRS* in both cell lines. *NRS* expression was considerably higher in C26 cells than in LLC cells (Figure 6A). Consistent with our data, a gene expression database of normal and tumor tissues from human samples (U133A datasets) showed that NRS is expressed at higher levels in colon cancer patients than in healthy controls, whereas no significant changes in NRS expression are observed in lung cancer patient samples (Supplementary Figure S4)^1^. To determine whether NRS also regulates the Hpo pathway in mammals, we examined the expression of YAP, a mammalian homolog of Yki, target genes in the two cell lines. YAP target genes were significantly upregulated in the C26 cell line, which has high expression of NRS (Figure 6B). Also, the upregulated YAP target gene level was suppressed by NRS inhibitor (TirB) treatment in C26 cells (Supplementary Figure S5A). In addition, the SAV1 protein level in C26 cells was significantly down-regulated compared to that in LLC cells (Supplementary Figure S5B), suggesting that upregulated NRS interacts with SAV1 to destabilize and inhibit Hpo signaling which is consistent to our fly data (Figures 5D,E). We then tested the effect of the NRS inhibitor TirB on tumor growth in mammalian cells. TirB had no effect in the LLC cell line (Figure 6C), whereas it significantly decreased cell proliferation in C26 cells (Figure 6D). Like NRS inhibition, YAP knockdown also showed reduced cell proliferation in C26 cells, but not in LLC cells (Supplementary Figures S5C–E), suggesting that NRS is required for YAP-induced proliferation in mammalian cells. Taken together, these results suggest that the modulation of NRS expression is critical for Hpo signaling-mediated tumorigenesis in different tumor types, providing a potential strategy to develop anticancer drugs targeting NRS.

## Discussion

NRS has been studied mostly for its role in translation, and there is little evidence of the non-canonical function of NRS in tumorigenesis. In the present study, we identified a novel role of NRS in growth regulation mediated by the Hpo signaling pathway. In a proposed model (Figure 6E), we suggest that NRS is mainly involved in translation under normal conditions, in which Hpo signaling is active and growth is regulated. Because the core components of the Hpo pathway (Hpo, Sav, and Wts) are tumor suppressors, functional mutation of the core components induces tumor growth (Pan, 2010; Zhao et al., 2010). We propose that, under these tumorigenic conditions, NRS is upregulated and physically interacts with Sav, thereby inhibiting Sav-Hpo binding. This interaction suppresses the Hpo-Wts-Yki phosphorylation cascade, promotes Yki nuclear localization, and activates Yki target genes, which are involved in growth regulation. We showed that TirB (NRS inhibitor) rescued cancer cell proliferation in *Drosophila* and mammals (Figures 2, 6). These findings suggest that NRS negatively regulates the Hpo-Sav-Wts cascade to induce cell proliferation.

The *Drosophila* NRS protein is well conserved with the mammalian system, sharing more than 80% sequence similarity. The proteins also share conserved domains, such as the aminoacyl synthetase-like catalytic core domain and the anticodon recognition domain, which are both critical for the canonical function of NRS. However, recent evidence supports that amino acid-binding sites are also essential for non-translational functions associated with signaling pathways such as mTORC1 activation through KRS (Han et al., 2012; Segev and Hay, 2012) and anti-apoptosis signaling through QRS (Ko et al., 2001). In some cases, such as the WHEP domain of WRS (Wakasugi et al., 2002) or the UNE-S domain of SRS (Xu et al., 2012), additional new domains that are not essential for catalytic activity regulate non-translational functions. A recent study suggests that the unique N-terminal extension domain of human NRS (UNE-N) interacts with CCR3, mediating proinflammatory signaling in interstitial lung disease (Park et al., 2018). Although we demonstrated a new non-translational function of NRS associated with tumorigenesis, the specific domain of NRS mediating the regulation of Hpo signaling was not identified. Future studies should identify the NRS domain involved in binding to Sav. We also showed that mammalian cancer cells with high expression of NRS regulate YAP target gene expression (Figure 6). This introduces the question of whether mammalian NRS also directly binds to SAV1, leading to the activation of YAP in the C26 cell line. Whether mammalian NRS regulates the Hpo signaling pathway through a mechanism similar to that in *Drosophila* remains to be determined.

Based on our results that NRS level was increased in yki overexpression flies (Supplementary Figure S1B) and this could raise the possibility that NRS plays a role in downstream of Yki. Many Hpo pathway components act as not only upstream regulators but also Yki target genes (e.g., *ex*, *wts*, *ft*, *ds*, and *kba*) for negative feedback loop. Similar to these components, there is a possibility that NRS might act as both upstream Hpo pathway component and Yki target gene for positive feedback loop.

The Hpo pathway is a key regulator of tumor development in both *Drosophila* and mammals. We identified NRS as a novel component of the Hpo pathway. A previous study identified KRS as a binding candidate of Wts, as demonstrated by mass spectrometry and co-IP (Kwon et al., 2013). These findings support the non-translational roles of ARSs in Hpo signaling-mediated tumorigenesis.

Studies in animal models led to the emergence of ARSs as potential therapeutic agents for the treatment of various diseases. For example, administration of glycyl-tRNA synthetase to colon cancer-bearing mice significantly suppresses tumor formation by dephosphorylating and deactivating ERK (Park et al., 2012). Our data indicated that the NRS inhibitor TirB rescued tumor growth in *Drosophila* as well as cell proliferation in mammalian cancer cells. NRS expression was high in colon carcinoma, but low in LLC cells (Figure 6A), supporting that NRS is expressed at higher levels in colon cancer patients, whereas no significant changes in NRS expression are observed in lung cancer patient samples from human database (Supplementary Figure S4). In addition, analysis of the human transcriptome shows that high expression of NRS in liver cancer is correlated with poor survival rates (Uhlen et al., 2017), suggesting that NRS expression can be used as a prognostic marker in certain cancer types.

Recently, small molecule inhibitors against PPIs have emerged as new anticancer drugs (Nero et al., 2014). For example, chemical or peptide inhibitors that block the PPI between p53 and MDM2 restore p53 function and can be used as anticancer drugs (Liu et al., 2010). Taken together, the present results suggest that the physical interaction of NRS and Sav is a potential target that may lead to the development of new anticancer drugs based on PPI inhibition.

## Data Availability Statement

All datasets generated for this study are included in the article/Supplementary Material.

## Author Contributions

EY, K-SL, and KY designed the research and wrote the manuscript. EY, D-WK, JL, and J-HL performed the experiments. EY, S-HK, K-JM, K-SL, and KY analyzed the data.

## Conflict of Interest

The authors declare that the research was conducted in the absence of any commercial or financial relationships that could be construed as a potential conflict of interest.

## Footnotes

1 http://medical-genome.kribb.re.kr/GENT

## Supplementary Material

FIGURE S1 Suppression of the tumor growth phenotype is NRS-specific. **(A)** *LeuRS-RNAi*, *MetRS-RNAi*, *PheRS-RNAi*, *TrpRS-RNAi*, and *ValRS-RNAi* did not suppress the tumor growth phenotype induced by *yki* overexpression. Scale bar, 200 μm. **(B)** *yki* overexpression upregulated *NRS* mRNA expression. **(C)** As a negative control, a cell proliferation marker was not significantly changed by *KRS-RNAi*. Blue, DAPI; green, GFP; red, phospho-H3. Scale bar, 50 μm. **(D)** Quantification of phospho-H3-positive cells in the GMR-expressing region. Data are presented as the mean ± SEM. ^∗^*p* < 0.05, ^∗∗^*p* < 0.01.

FIGURE S2 Cytotoxicity analysis of TirB. Analysis of lifespan in flies treated with TirB showed no cytotoxic effects in male **(A)** and female flies **(B)**. **(C)** *yki* overexpression increased the phosphorylation activity of S6K. NRS knockdown reduced the phosphorylation activity of S6K in the *yki-*overexpression background. **(D)** Quantification of the intensity of western blot bands in panel **(C)**. Data are presented as the mean ± SEM. ^∗^*p* < 0.05, ^∗∗^*p* < 0.01.

FIGURE S3 Genetic interaction between NRS and Sav. **(A)** *ey* > *Gal4/*+ was used as the control, *n* = 6. **(B)** *Sav* overexpression resulted in slightly smaller eye size, *n* = 7. **(C)** *NRS* overexpression resulted in bigger eye size *n* = 7. **(D)** Co-expression of *NRS* and *Sav* rescued the NRS-induced enlarged eye *n* = 8. Scale bar, 200 μm. **(E)** Quantification of relative eye size from panels **(A–D)**. Co-IP assay showed that NRS did not directly bind to Hpo **(F)** and Yki **(G)**. **(H)** Quantification of Sav input protein levels in Figure 5F. **(I)** Overexpression of Hpo suppressed the overgrowth eye phenotype of *GMR* > *yki^*S*168*A*^.* Data are presented as the mean ± SEM. ^∗^*p* < 0.05, ^∗∗^*p* < 0.01.

FIGURE S4 A gene expression database of normal and tumor tissues from human samples. A gene expression database across normal and tumor tissue (U133A datasets) (http://medical-genome.kribb.re.kr/GENT) showed that NRS is highly expressed in colon cancer patients than in healthy controls, but not in lung cancer patient samples (C – cancer tissue, N – normal tissue).

FIGURE S5 YAP target gene expression by NRS inhibition and cell proliferation assay by YAP knockdown. **(A)** Increased YAP target gene (*BIRC5*) level in C26 cell line was reduced by TirB treatment, but no reduction was observed in LLC cell line. **(B)** SAV1 protein expression level was decreased in C26 cells compared to LLC cells. **(C)** YAP-siRNA was efficiently knocked down in C26 cells. **(D,E)** Cell proliferation was reduced by YAP knockdown in C26 cells **(D)**, but not in LLC cells **(E)**. Data are presented as the mean ± SEM. ^∗^*p* < 0.05, ^∗∗^*p* < 0.01.
