# Supplementary material for: Asparaginyl-tRNA Synthetase, a Novel Component of Hippo Signaling, Binds to Salvador and Enhances Yorkie-Mediated Tumorigenesis
Source: Front Cell Dev Biol. 2020 Feb 5;8:32. doi: 10.3389/fcell.2020.00032 (PMC7014954; doi:10.3389/fcell.2020.00032)

Supplemental Figure 1

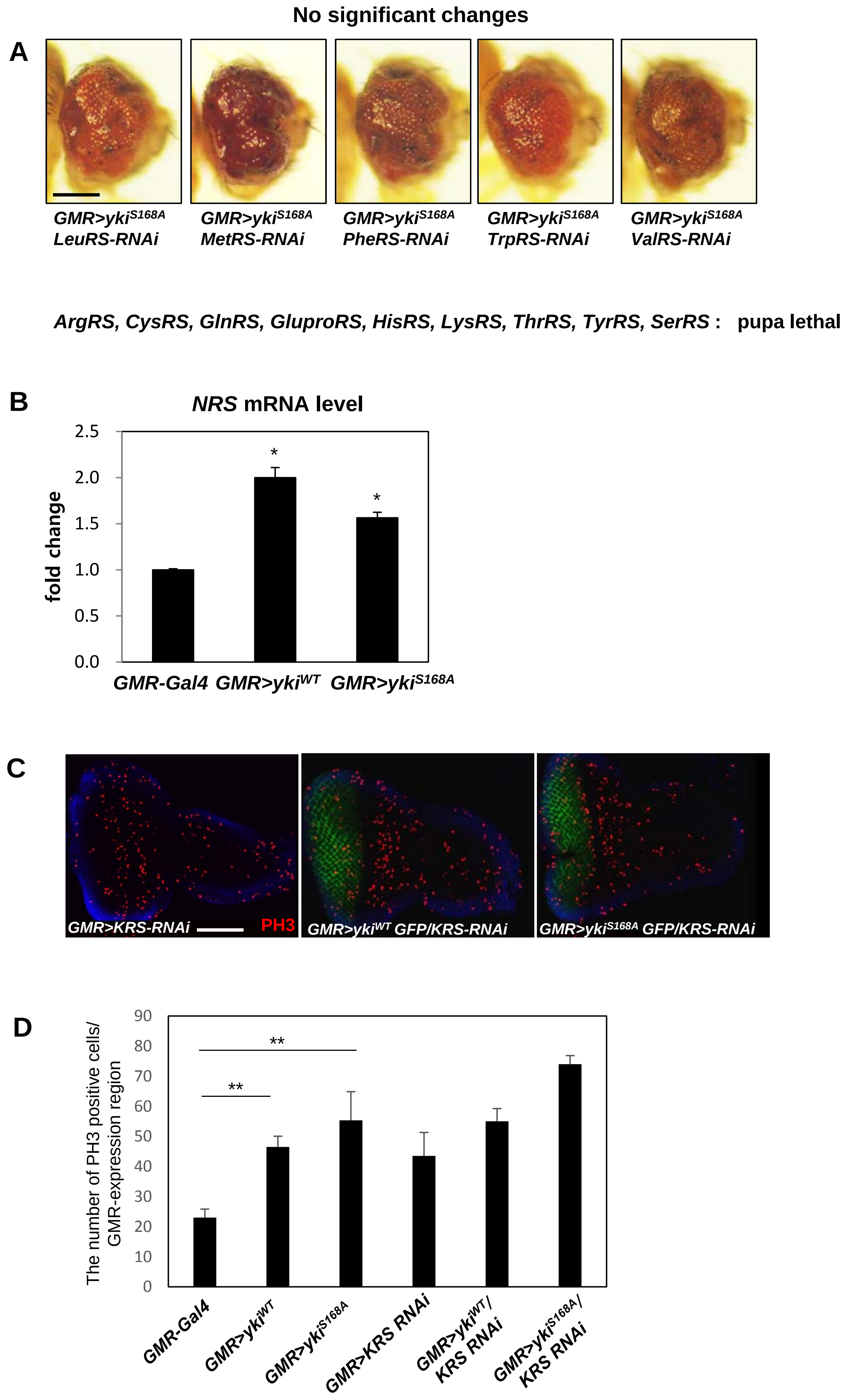

Supplemental figure 2

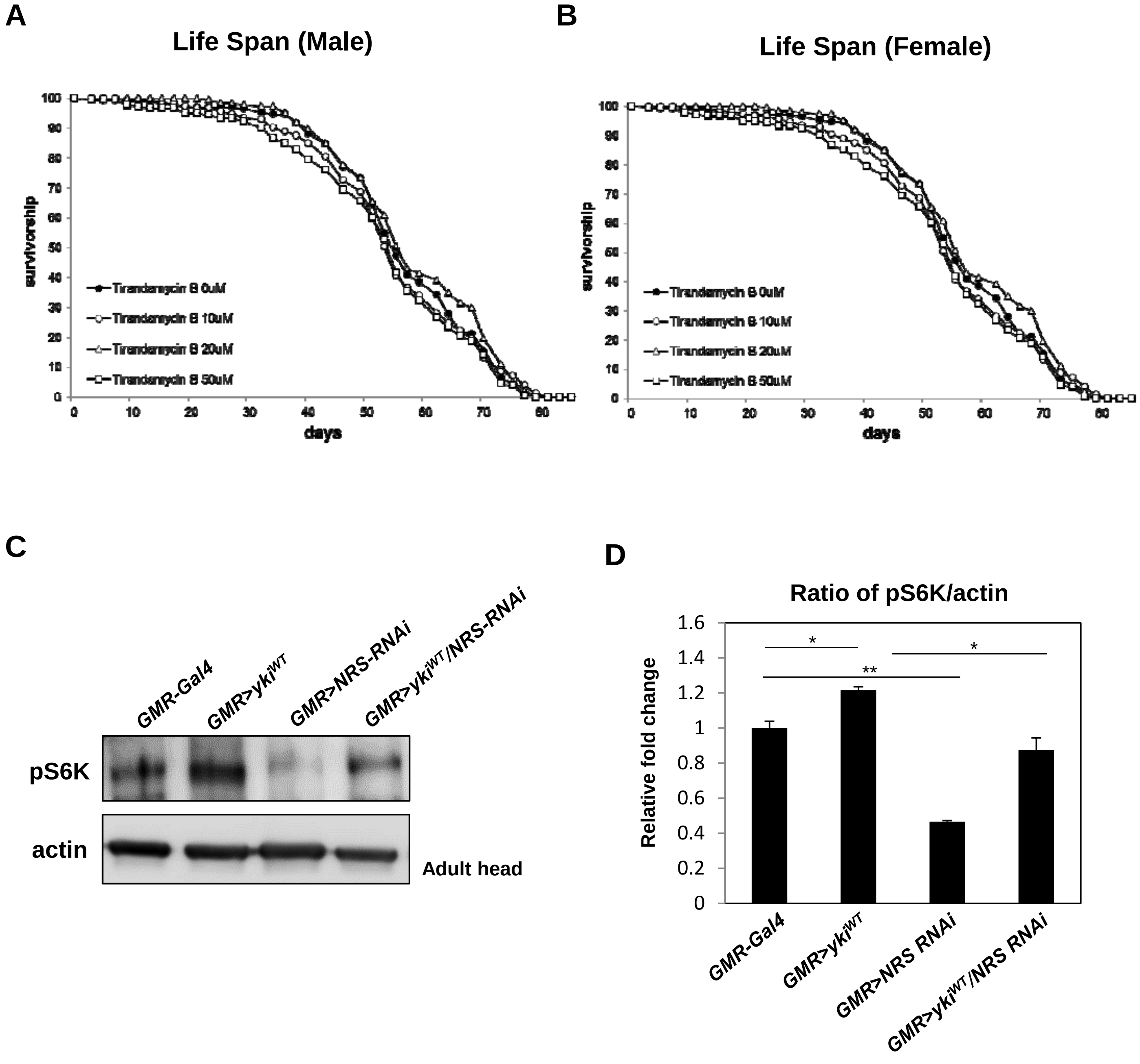

Supplemental Figure 3

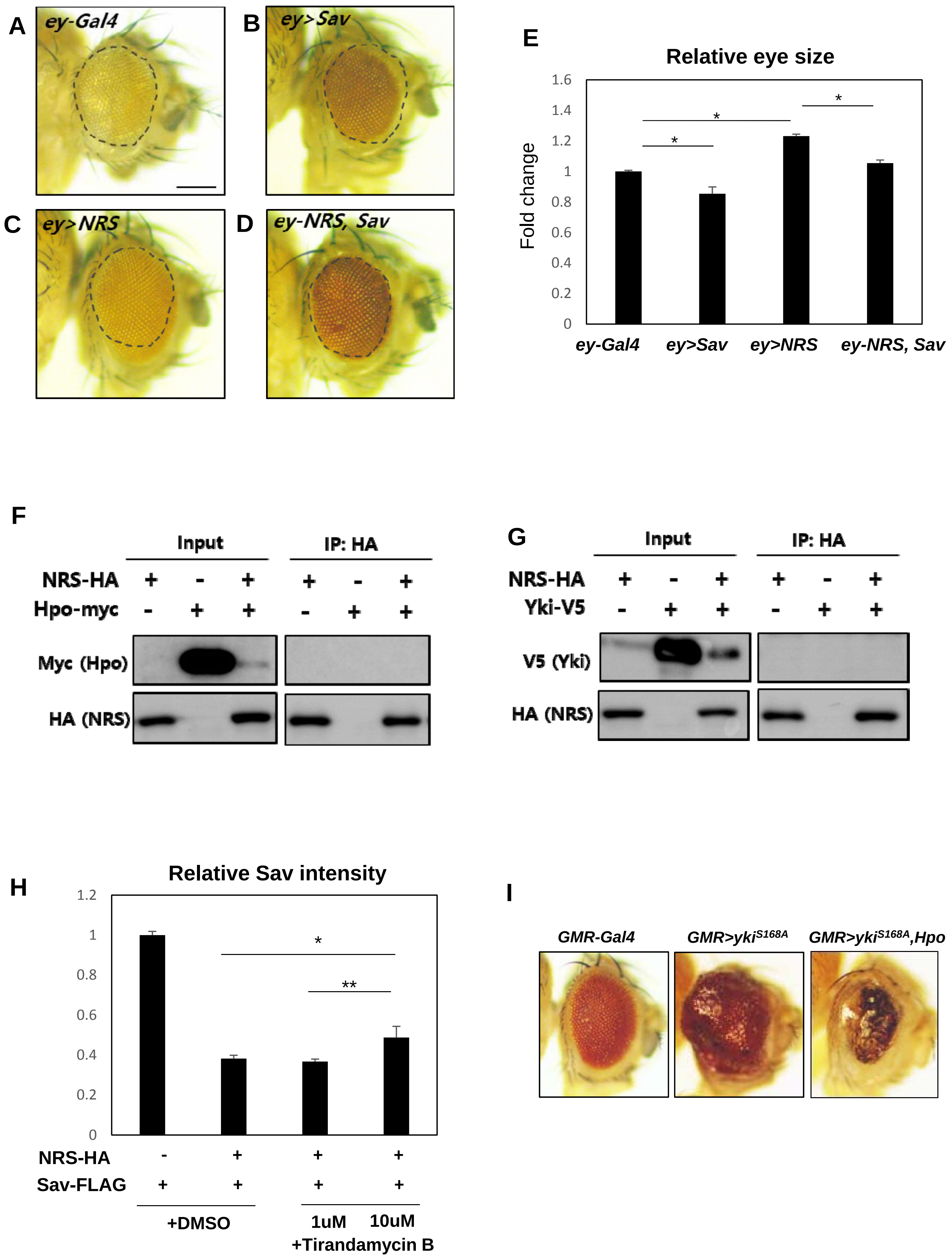

Supplemental Figure 4

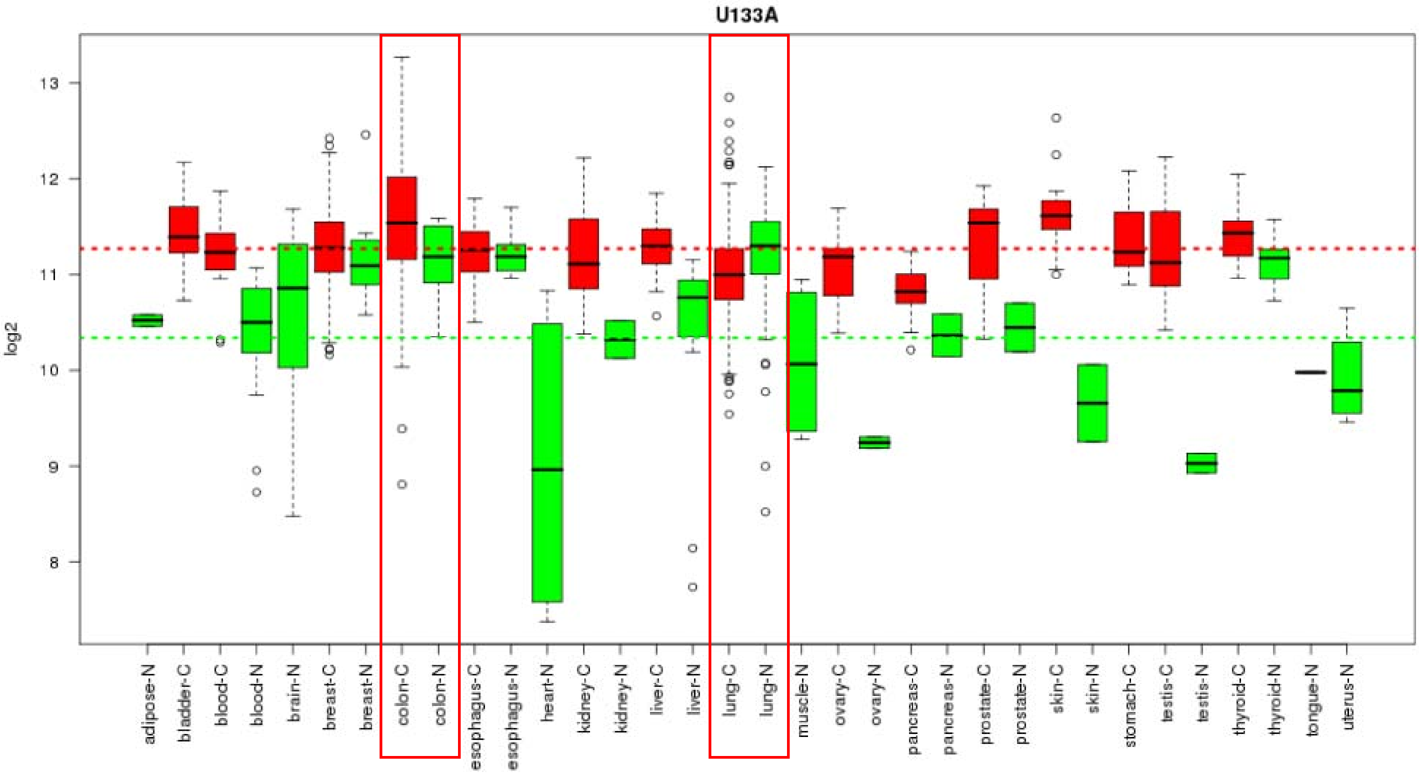

Supplemental Figure 5

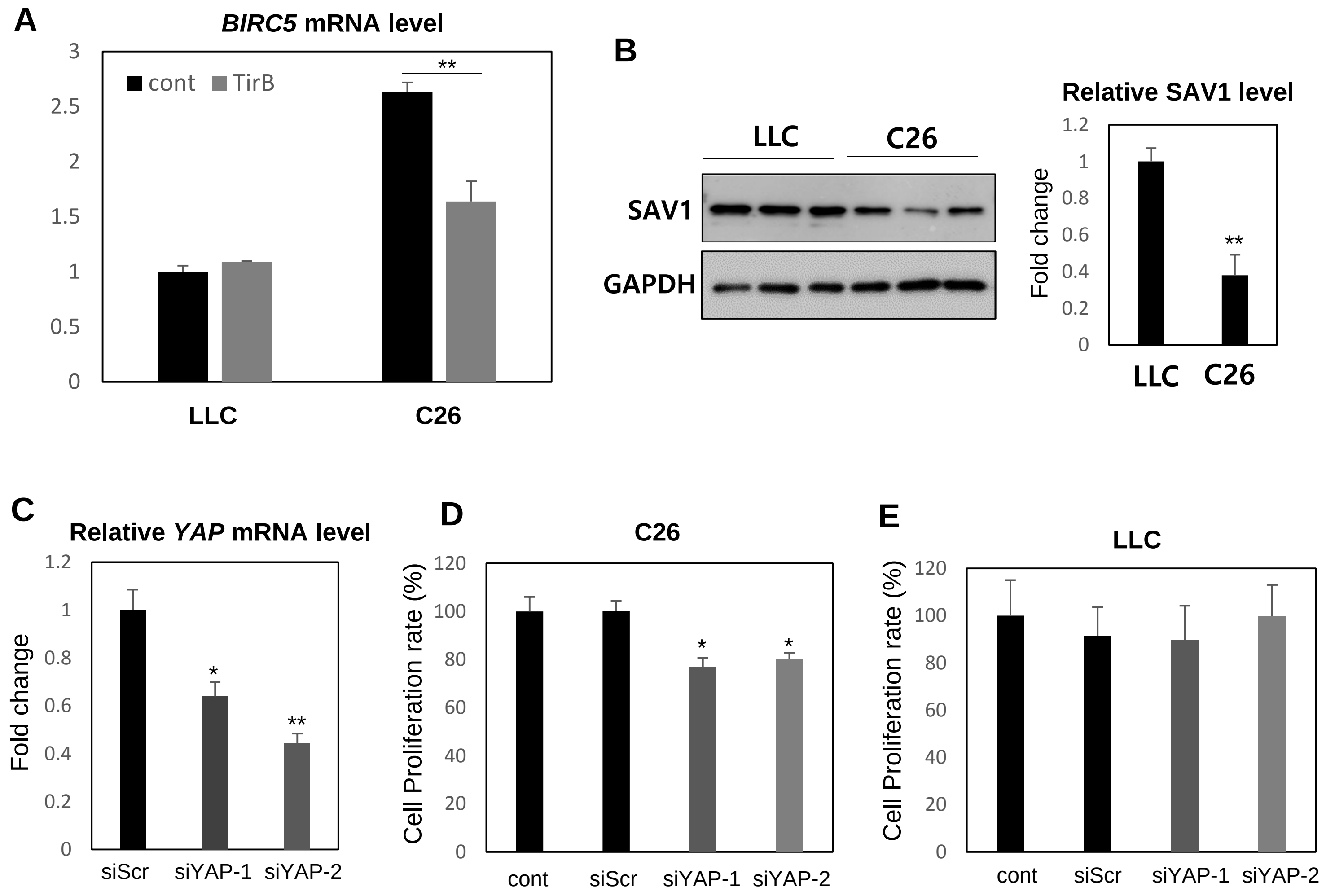

Supplement: FIGURE S1 — Suppression of the tumor growth phenotype is NRS-specific. (A) LeuRS-RNAi, MetRS-RNAi, PheRS-RNAi, TrpRS-RNAi, and ValRS-RNAi did not suppress the tumor growth phenotype induced by yki overexpression. Scale bar, 200 μm. (B) yki overexpression upregulated NRS mRNA expression. (C) As a negative control, a cell proliferation marker was not significantly changed by KRS-RNAi. Blue, DAPI; green, GFP; red, phospho-H3. Scale bar, 50 μm. (D) Quantification of phospho-H3-positive cells in the GMR-expressing region. Data are presented as the mean ± SEM. ∗p < 0.05, ∗∗p < 0.01. [file Data_Sheet_1.PDF]
